# Inducible Gene Manipulations in Brain Serotonergic Neurons of Transgenic Rats

**DOI:** 10.1371/journal.pone.0028283

**Published:** 2011-11-29

**Authors:** Tillmann Weber, Kai Schönig, Björn Tews, Dusan Bartsch

**Affiliations:** 1 Department of Molecular Biology, Central Institute of Mental Health, Mannheim, Germany; 2 Department of Addictive Behavior and Addiction Medicine, Central Institute of Mental Health, Mannheim, Germany; 3 Department of Psychiatry and Psychotherapy, Central Institute of Mental Health, Mannheim, Germany; 4 Brain Research Institute, ETH Zürich, Zürich, Switzerland; 5 Medical Faculty Mannheim, Heidelberg University, Mannheim, Germany; Louisiana State University Health Sciences Center, United States of America

## Abstract

The serotonergic (5-HT) system has been implicated in various physiological processes and neuropsychiatric disorders, but in many aspects its role in normal and pathologic brain function is still unclear. One reason for this might be the lack of appropriate animal models which can address the complexity of physiological and pathophysiological 5-HT functioning. In this respect, rats offer many advantages over mice as they have been the animal of choice for sophisticated neurophysiological and behavioral studies. However, only recently technologies for the targeted and tissue specific modification of rat genes - a prerequisite for a detailed study of the 5-HT system - have been successfully developed. Here, we describe a rat transgenic system for inducible gene manipulations in 5-HT neurons. We generated a Cre driver line consisting of a tamoxifen-inducible CreERT2 recombinase under the control of mouse *Tph2* regulatory sequences. Tissue-specific serotonergic Cre recombinase expression was detected in four transgenic TPH2-CreERT2 rat founder lines. For functional analysis of Cre-mediated recombination, we used a rat Cre reporter line (CAG-loxP.EGFP), in which EGFP is expressed after Cre-mediated removal of a loxP-flanked lacZ STOP cassette. We show an in-depth characterisation of this rat Cre reporter line and demonstrate its applicability for monitoring Cre-mediated recombination in all major neuronal subpopulations of the rat brain. Upon tamoxifen induction, double transgenic TPH2-CreERT2/CAG-loxP.EGFP rats show selective and efficient EGFP expression in 5-HT neurons. Without tamoxifen administration, EGFP is only expressed in few 5-HT neurons which confirms minimal background recombination. This 5-HT neuron specific CreERT2 line allows Cre-mediated, inducible gene deletion or gene overexpression in transgenic rats which provides new opportunities to decipher the complex functions of the mammalian serotonergic system.

## Introduction

5-hydroxytryptamine (5-HT, serotonin) has been implicated in a wide variety of emotional, cognitive and behavioral processes. Psychopharmacotherapeutic agents targeting molecules of the serotonergic system are often used for the treatment of a wide spectrum of psychiatric disorders. Although this clearly demonstrates the functional relevance of 5-HT for physiological as well as disease processes, there is no well-defined framework for comprehending any of its roles [1].

The understanding of the 5-HT system's function and its underlying molecular mechanisms has been strongly accelerated by using reverse genetic approaches in transgenic mouse models [2]–[11]. However, in mice the analysis of certain phenotypes reaches its limits, as complex behavioral tasks involving higher order cognitive functions are difficult to perform. Indeed, most behavioral and electrophysiological studies are traditionally conducted in rats and therefore many behavioral tests are only validated for this species. The rat behavioral repertoires and the related neural correlates have been well described and physiological interventions, microsurgery and toxicology studies as well as evaluation of higher order functions are in general more sophisticated and informative in rats than in mice [12]. As a consequence, most of the research on serotonergic functioning has been accomplished using rats despite the fact that only few rats with specific genetic manipulations of the 5-HT system are available [13].

Recently, it has become feasible to manipulate the rat's genome with conditional transgenesis [14]. In the near future, technological advances in the rat such as zinc finger nucleases [15] and the development of germline competent rat embryonic stem cells [12] will enable researchers to spatially and temporally control gene manipulation. For this purpose, it will be necessary to control gene expression or gene deletion with tissue-specific Cre-driver lines which allow the recombination of loxP-flanked target sequences in the rat genome. To specifically manipulate target genes within the serotonergic system, Cre drivers could be linked to regulatory sequences of 5-HT neuron specific genes such as *Pet-1* or *Tph2*.

In the present study, we generated and characterized four transgenic TPH2-CreERT2 rat lines in which a 177 kb genomic sequence of the mouse *Tph2* gene controls tissue-specific expression of the CreERT2 recombinase. Cre-mediated recombination of loxP flanked target genes was functionally characterized with the Cre reporter line pCaggs-loxP.lacZ.loxP-EGFP (CAG-loxP.EGFP). After tamoxifen treatment of double transgenic TPH2-CreERT2/CAG-loxP.EGFP rats, efficient EGFP expression and hence recombination occured specifically in 5-HT neurons while background recombination in the absence of tamoxifen could not be identified.

## Methods

### Generation of TPH2-CreERT2 transgenic rats

A PAC (L065) which contains the full-length mouse *Tph2* gene (107 kb) with 51 kb upstream and 19 kb downstream DNA sequences was modified as previously described [16]. The purified, linearized TPH2-CreERT2 DNA was microinjected into the pronucleus of oocytes of Sprague-Dawley rats (Charles River Laboratories, Germany). Transgenic founder rats were identified by PCR genotyping of tail tips. The TPH2-CreERT2 transgenic rats were bred with the Cre reporter line CAG-loxP.EGFP (Schönig et al, in preparation) to generate double-transgenic TPH2-CreERT2/CAG-loxP.EGFP rats. In brief CAG-loxP.EGFP rats harbour a loxP-flanked lacZ reporter gene, controlled by the ubiquitously active CAG promoter [17], [18]. The lacZ DNA fragment precludes the transcription of a second reporter gene EGFP. Cre mediated recombination can be monitored in double-transgenic TPH2-CreERT2/CAG-loxP.EGFP by EGFP expression.

### Quantification of transgene copy number

Copy number quantification of the TPH2-CreERT2 transgene per cell was done via genomic quantitative real-time PCR (qPCR) for each TPH2-CreERT2 line. For amplification and data collection, we used the Rotor-Gene Q-system (Qiagen). All reactions were carried out in a total volume of 25 µL and were measured in triplicates. Each reaction mixture contained 5 ng of genomic DNA, 12.5 µl Rotor-Gene Fast SYBR Green Master Mix (Qiagen) and 300 nM forward and reverse primers. The amplification protocol consisted of an initial denaturation step at 95°C for 5 min, followed by 40 cycles at 95°C for 10 s, 60°C for 10 s and 72°C for 10 s. SYBR Green fluorescence was detected at 72°C. Each amplification reaction was checked for the absence of nonspecific PCR products by melting curve analysis followed by agarose gel electrophoresis.

The absolute target copy numbers were determined using 1∶2 dilution series of genomic mouse DNA harbouring defined numbers of Cre transgenes [19] as an external standard. For each sample, the amount of Cre transgene and reference gene (ApoB) was measured in each transgenic line. The following primers were used: Cre3: 5′ TCG CTG CAT TAC CGG TCG ATG C 3′; Cre4: 5′ CCA TGA GTG AAC GAA CCT GGT CG 3′; ApoB_for: 5′ ATC TCA GCA CGT GGG CTC 3′; ApoB_rev 5′ TCA CCA GTC ATT TCT GCC TTT G 3′.

### In vivo induction of Cre-mediated recombination with tamoxifen

Tamoxifen (Sigma) was dissolved in neutral oil at a final concentration of 20 mg/ml. For recombination analysis, double-transgenic TPH2-CreERT2/CAG-loxP.EGFP rats (8–12 weeks) were given a protocol of alternating daily tamoxifen injections (40 mg/kg) for a total of five consecutive days. The protocol was designed with single injections on days 1, 3 and 5 and two tamoxifen injections twelve hours apart on days 2 and 4. Control animals were injected with neutral oil (vehicle) using the same schedule. Rats were sacrificed 14 days after the last injection.

All experimental procedures were approved by the local Animal Welfare Committee (Regierungspräsidium Karlsruhe 35-918581/G-107/09) and carried out in accordance with the local Animal Welfare Act and the European Communities Council Directive of 24 November 1986 (86/609/EEC).

### Immunohistochemistry

Transgenic TPH2-CreERT2 founder rats were characterized by immunohistochemistry using DAB staining (Vectastain Elite ABC kit) with a rabbit α-Cre primary antibody (Covance, 1∶2500). Founder line #15 was further characterized with dual-label fluorescent immunohistochemistry in TPH2-CreERT2 and TPH2-CreERT2/CAG-loxP.EGFP rats. The following primary antibodies were used: chicken α-βgalactosidase (Abcam, 1∶10000), rabbit α-GFP (Invitrogen, 1∶1000), rabbit α-Cre (Covance, 1∶1000), mouse α-GAD67 (Millipore, 1∶500), mouse α-TH (Millipore, 1∶500), mouse α-NeuN (Millipore, 1∶4000), mouse α-GFAP (Sigma, 1∶2000), rabbit α-TPH2 (Dianova, 1∶5000), and mouse α-TPH1 (Sigma, 1∶2000) antibodies. Tryptophane hydroxylase 2 (TPH2) is the rate-limiting enzyme of 5-HT synthesis in the brain and specific to serotonergic neurons. The anti-tryptophan hydroxylase 1 (TPH1) antibody crossreacts with TPH2 and detects both isoenzymes. Secondary antibodies were AF488 donkey α-rabbit (Invitrogen, 1∶1000 for TPH2 and 1∶5000 for GFP and Cre), Cy3 donkey α-mouse (Jackson ImmunoResearch, 1∶200 for TPH1), Cy3 donkey α-chicken (Jackson ImmunoResearch, 1∶1000 for βgalactosidase) and AF488 donkey α-mouse (Invitrogen, 1∶200 for GAD67, NeuN, TH and GFAP). Sections were examined using a Nikon C1Si-CLEM confocal laser-scanning microscope (Nikon Imaging Center, BioQuant, Heidelberg, Germany). Confocal image stacks for both channels were acquired sequentially, and projected on average using ImageJ software.

### Statistical methods

Coronal slices of 3 adult TPH2-CreERT2/CAG-loxP.EGFP rats (8–10 weeks old) per group were processed with dual-label fluorescent immunohistochemistry detecting GFP and TPH. Image stacks of slices that showed TPH staining were acquired using a confocal laser-scanning microscope. The ratio of GFP+/TPH+ neurons to all TPH+ neurons was calculated separately for caudal, median and dorsal raphe nuclei. Confidence-bounds (CI) for recombination efficacy and background recombination in adult rats were calculated using the Clopper-Pearson method based on significance level 95%.

## Results

### Generation of TPH2-CreERT2 transgenic rats

**Figure 1:**
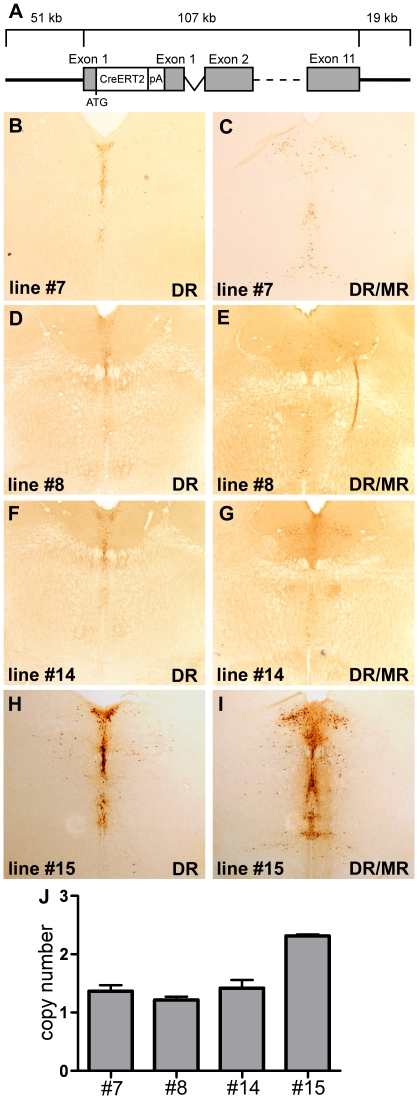
Copy number dependent Cre expression in TPH2-CreERT2 founder rats. (A) Mouse TPH2-CreERT2 expression cassette for DNA microinjection. (B–I) DAB-immunohistochemistry with a Cre antibody shows weak Cre expression in the brain stem and mid-brain of TPH2-CreERT2 rat founder lines #7 (Fig. 1B,C), #8 (Fig. 1D,E), and #14 (Fig. 1F,G). Founder line #15 shows extensive Cre staining in areas where serotonergic raphe nuclei are located (Fig. 1H,I). Intensity of Cre expression correlates with the transgene copy number of TPH2-CreERT2 rat founders (Fig. 1J).

For inducible, tissue-specific expression of CreERT2 in serotonergic neurons of the rat brain, a 177 kb fragment of mouse genomic DNA containing the *Tph2* gene and its regulatory elements was used [16]. The linearized CreERT2 expression cassette (Fig. 1A) was introduced into the rat genome via pronuclear microinjection of fertilized Sprague Dawley rat oocytes. Seven transgenic founder rats were identified by PCR of tail DNA. Of those, three founders did not transmit their transgene leaving four founders for characterization.

### Cre expression in TPH2-CreERT2 founder rats

**Figure 2:**
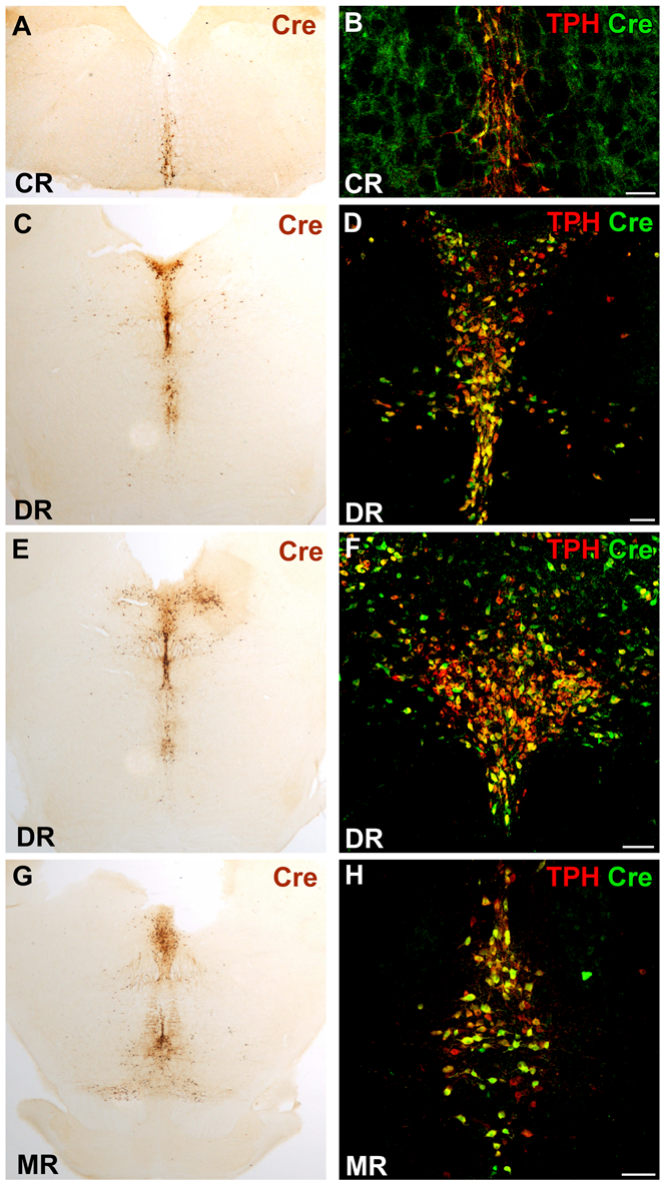
Cre expression is restricted to serotonergic neurons of the raphe nuclei. (A,C,E,G) DAB-immunohistochemistry with a Cre antibody of line #15 shows Cre staining in the brain stem and mid-brain, regions which contain serotonergic somata while extraserotonergic brain regions show no staining. (B,D,F,H) Coronal sections of dual-label fluorescence immunohistochemistry with Cre and TPH1 antibodies. The TPH1 antibody crossreacts with TPH2 and detects both isoenzymes. Colocalization of TPH1 and Cre confirms exclusive Cre expression in 5-HT neurons of the raphe nuclei. Caudal raphe nuclei (CR); dorsal raphe nuclei (DR); median raphe nuclei (MR). Scale bars: 100 µm.

All four TPH2-CreERT2 founder lines showed Cre immunostaining in the raphe nuclei of the brain stem and midbrain while no Cre expression was observed outside the raphe nuclei. The efficacy of Cre expression varied notably among the founder lines. The TPH2-CreERT2 founder lines #7, #8 and #14 showed weak and mosaic Cre staining in serotonergic neurons (Fig. 1B–G) while strong Cre expression could be detected in the raphe nuclei of line #15 (Fig. 1H,I). Since large genomic DNA constructs are thought to regulate transgene expression independent of their integration site but copy number dependent [20], we determined the transgene copy number of each TPH2-CreERT2 founder by qPCR (Fig. 1J). Stronger Cre expression in founder line #15 could be correlated with increased transgene copy number while the weakly expressing founder lines #7, #8 and #14 contained only a single copy of the transgene. Cre expression in founder line #15 (Fig. 2A,C,E,G) was further investigated for their tissue-specificity with dual-label fluorescent immunohistochemistry using a 5-HT neuron specific TPH antibody and a Cre antibody (Fig. 2B,D,F,H). Colocalization of Cre and TPH demonstrated that Cre was exclusively expressed in 5-HT neurons. Hence, the transgenic rat founder line #15 showed extensive and tissue-specific Cre expression in 5-HT neurons of all raphe nuclei.

### Inducible and efficient recombination in serotonergic neurons of double-transgenic TPH2-CreERT2/CAG-loxP.EGFP rats

Line #15 was further used to functionally characterize the temporal and spatial control of tamoxifen-induced CreERT2-mediated recombination in 5-HT neurons. We made use of a rat Cre reporter line (CAG-loxP.EGFP), which has been shown to efficiently monitor Cre-mediated recombination in forebrain principal neurons (Schönig et al, in preparation). Here, the ubiquitously active CAG-promoter [17], [18], [21] drives the expression of a double reporter. Under uninduced baseline conditions, the loxP-flanked lacZ minigene is expressed, reflecting cell-type specific CAG-promoter activity. Upon Cre-mediated recombination, lacZ is replaced with the second reporter gene enhanced green fluorescent protein (EGFP). The appearance of EGFP serves as an indicator of Cre mediated recombination in double transgenic rats.

**Figure 3:**
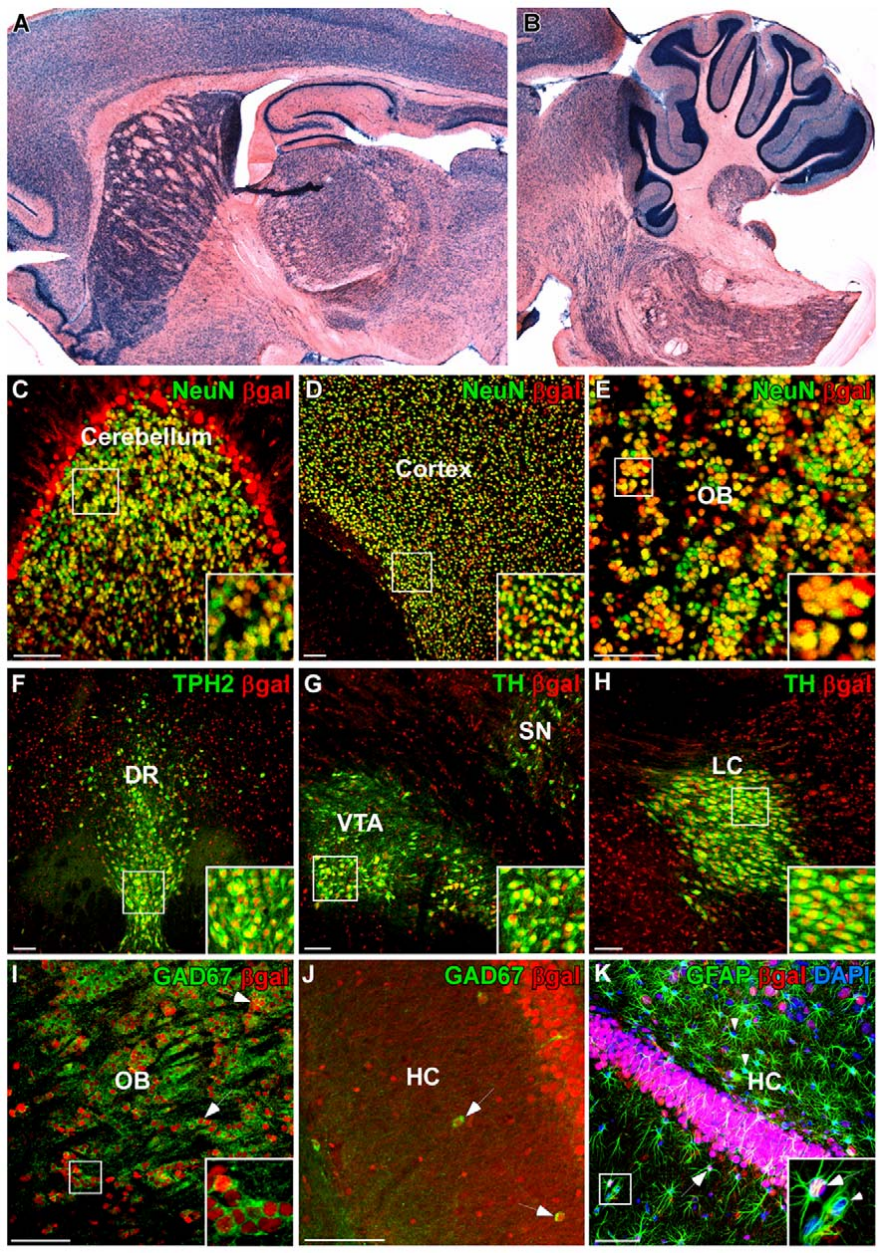
Baseline βgal expression in the brain of CAG-loxP.EGFP Cre reporter rats. (A,B) X-Gal staining of sagittal sections shows ubiquitous βgal activity throughout the brain of adult CAG-loxP.EGFP rats (P90). (C–K) Dual-label fluorescence immunohistochemistry (IHC). (C–E) βgal/NeuN IHC of the cerebellum (C), cortex (D) and OB (E) shows strong colocalization of βgal with the neuronal marker NeuN. (F–H) βgal IHC with the serotonergic marker TPH2 (F), and the dopaminergic and noradrenergic marker tyrosine hydroxylase (TH) (G,H) shows abundant colocalization of βgal with 5-HT neurons in the dorsal raphe (F), with dopaminergic neurons in the ventral tegmental area and substantia nigra (G) and noradrenergic neurons in the locus coeruleus (H) confirming strong βgal expression in all monoaminergic neurons. (I,J) βgal/GAD67 IHC shows βgal expression in GABAergic neurons of the granular layer of the OB (I) and in the hippocampus (J). (K) βgal/GFAP IHC in the hippocampus shows infrequent βgal expression in glia. OB, olfactory bulb; DR, dorsal raphe nuclei; VTA, ventral tegmental area; SN, substantia nigra; LC, locus coeruleus; HC, hippocampus. Scale bars: 100 µm.

A prerequisite for a versatile Cre reporter line is the ability to monitor recombination in a wide range of cells. We first analysed baseline expression of beta-galactosidase (βgal) in serotonergic neurons and other cell types of the brain by dual-label fluorescent immunohistochemistry to determine expression characteristics of the CAG-loxP.EGFP line. CAG-driven βgal expression was found in virtually all brain regions (Fig. 3A–E) and in many types of neurons including monoaminergic (Fig. 3F–H) and GABAergic neurons (Fig. 3I–J). In contrast to neuronal expression, βgal expression was only infrequently found in astrocytes (Fig. 3K). These results verify the utility of the CAG-loxP.EGFP reporter line for monitoring Cre-mediated recombination not only in serotonergic neurons, but also in other neuronal subtypes.

**Figure 4:**
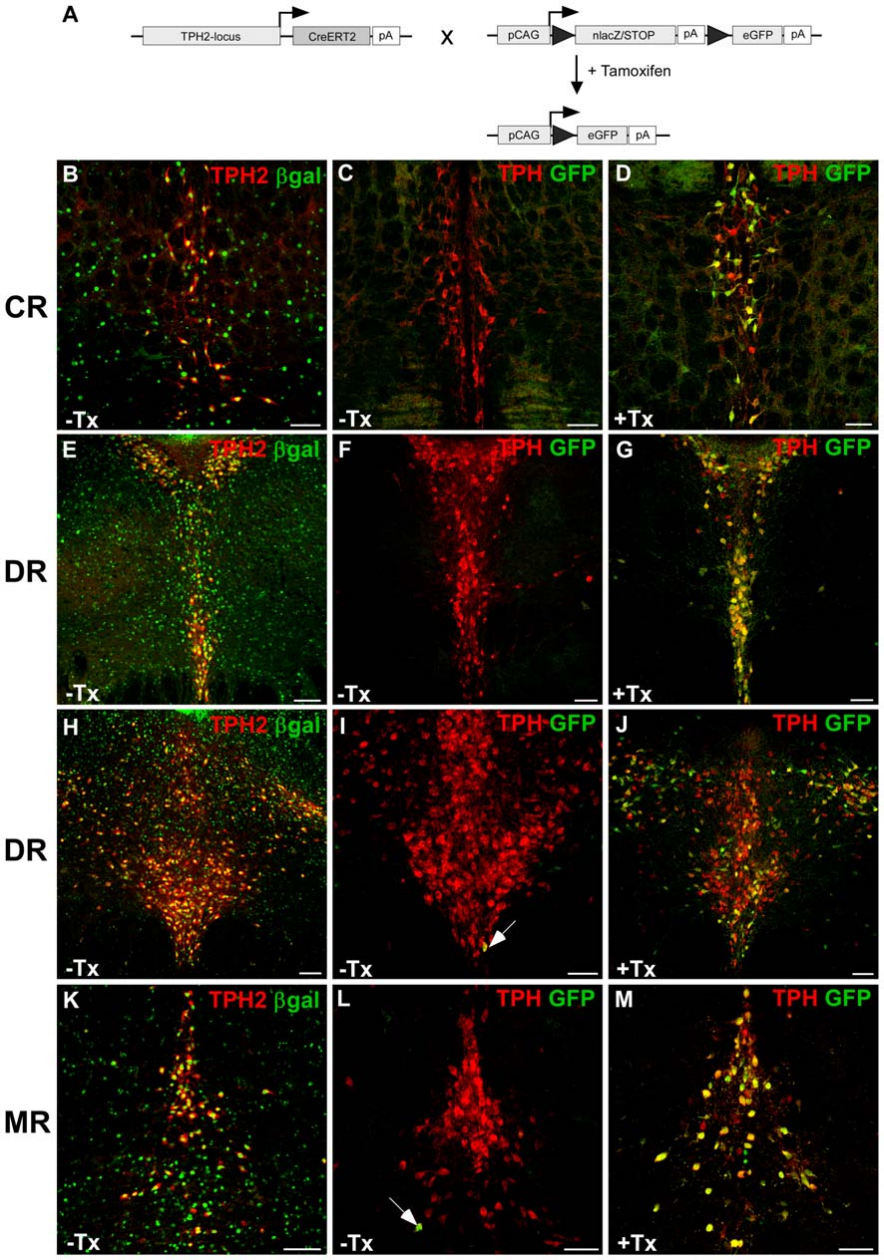
Inducible recombination is restricted to serotonergic neurons of adult TPH2-CreERT2/CAG-loxP.EGFP rats. (A) TPH2-CreERT2 rats were bred to CAG-loxP.EGFP rats to generate double-transgenic TPH2-CreERT2/CAG-loxP.EGFP rats. Under uninduced baseline conditions, the loxP-flanked lacZ minigene is expressed reflecting cell-type specific CAG-promoter activity. Upon Cre-mediated recombination (+ Tamoxifen), lacZ is replaced with the second reporter gene enhanced green fluorescent protein (EGFP). The appearance of EGFP serves as an indicator of Cre mediated recombination in double transgenic rats. TPH2-CreERT2/CAG-loxP.EGFP rats were daily injected with tamoxifen (40 mg/kg) or vehicle for five consecutive days starting between P60–90. Coronal sections show dual-label fluorescence immunohistochemistry for TPH/βgal (B,E,H,K) and TPH/GFP (C,F,I,L) in vehicle-treated rats (-Tx) and TPH/GFP in tamoxifen-treated (+Tx) rats (D,G,J,M). Colocalization is visualized at the level of caudal raphe nuclei (CR) (B–D), dorsal raphe nuclei (DR) (E–J) and median raphe nuclei (MR) (K–M) using confocal images. In vehicle-treated rats, TPH2-CreERT2/CAG-loxP.EGFP rats display strong basal, non-recombined βgal expression in TPH2+ 5-HT neurons (B,E,H,K) making these rats ideally suited to monitor tamoxifen-induced Cre-mediated recombination in 5-HT neurons. (C,F,I,L) Without tamoxifen treatment, background recombination, i.e. EGFP expression (arrows) hardly occurs. (D,G,J,M) After tamoxifen treatment, the majority of TPH+ 5-HT neurons in all raphe nuclei now show EGFP expression indicating Cre-mediated recombination in these neurons (GFP+/TPH+). Scale bars: 100 µm.

Based on these results, we generated double-transgenic TPH2-CreERT2/CAG-loxP.EGFP rats to determine recombination efficiency and tissue specificity for our rat Cre driver line TPH2-CreERT2 (Fig. 4A). Coronal brain sections from tamoxifen and vehicle treated TPH2-CreERT2/CAG-loxP.EGFP rats were analysed using dual-label fluorescent immunohistochemistry with βgal/TPH2 and GFP/TPH1 antibodies. In vehicle treated TPH2-CreERT2/CAG-loxP.EGFP rats, βgal-expression could be detected in virtually all 5-HT neurons (Fig. 4B,E,H,K). In contrast, EGFP expression could only be detected in few 5-HT neurons (Fig. 4C,F,I,L), which indicates minimal Cre-mediated background recombination in the absence of tamoxifen (Table 1). Importantly, in tamoxifen-treated TPH2-CreERT2/CAG-loxP.EGFP rats, EGFP and TPH expression colocalised in 5-HT neurons of caudal, dorsal and median raphe nuclei indicating effective Cre-mediated recombination in all raphe nuclei (Fig. 4D,G,J,M; Table 1). Extra-serotonergic brain regions showed no EGFP staining.

**Table 1:** Recombination efficacy and background recombination for rat TPH2-CreERT2 line #15.

	Line	absolute recombination frequency (GFP+/TPH+):TPH+	relative recombination frequency	95% CI
**+ Tamoxifen**	#15 CR	145∶195	74%	68–80%
	#15 DR	1787∶2368	75%	74–77%
	#15 MR	751∶937	80%	78–83%
	#15 total	2683∶3500	77%	75–78%
**+ Vehicle**	#15 CR	2∶175	1.1%	0.0–2.7%
	#15 DR	9∶1921	0.5%	0.2–0.8%
	#15 MR	8∶506	1.6%	0.05–3.4%
	#15 total	19∶2602	0.7%	0.4–1.1%

Caudal (CR), median (MR) and dorsal (DR) raphe nuclei were separately and jointly (total) calculated. Confidence-bounds (CI) were calculated using the Clopper-Pearson method based on significance level 95.0%.

## Discussion

In this study, we describe an inducible, tissue-specific rat transgenic CreERT2 driver line for conditional gene manipulations in serotonergic neurons. We functionally demonstrate efficient, tamoxifen-inducible, 5-HT neuron specific recombination with minimal background activity in TPH2-CreERT2 rats crossed to the rat Cre reporter line pCAG-loxP.EGFP.

### Application of mouse genomic regulatory sequences for the generation of tissue-specific rat Cre driver lines

TPH2 is the rate-limiting enzyme of 5-HT synthesis and strongly and exclusively expressed in serotonergic neurons of the raphe nuclei in the brain [22]. Hence, regulatory elements of *Tph2* should be suitable to direct Cre expression specifically to 5-HT neurons. We previously made use of large regulatory elements of the *Tph2* locus identified on a genomic mouse PAC clone to generate a TPH2-CreERT2 mouse line that shows highly efficient, tamoxifen-inducible recombination in 5-HT neurons [16]. As not only the rat and mouse *Tph2* genes are almost identical [22], but also the entire region of the mouse *Tph2* locus on chromosome 10 is highly homologous to the rat locus on 7q22 (NCBI Blast), we decided to use the same 177 kb TPH2-CreERT2 construct to generate transgenic rats. We demonstrate the fidelity of the mouse *Tph2* locus to direct Cre expression selectively to serotonergic neurons in transgenic rats. With this TPH2-CreERT2-expression cassette, transgenic 5-HT neuron-specific Cre expression is likely not dependent on the genomic site of integration since all founder lines showed Cre expression in the raphe nuclei. More likely, the efficacy of *Tph2*-controlled Cre expression appears to depend on the transgenic copy number. This is in accordance with previous reports showing that large genomic DNA constructs allow copy-number dependent transgene expression independent of the genomic integration site of the construct [20], [23], [24]. Efficient serotonergic Cre expression could only be found in founder line #15 which contained 2–3 transgene copies compared to single copies in all other founder lines.

Interestingly, we could not identify founder lines with higher copy numbers [24]. It remains to be investigated whether this finding of low copy numbers in rat transgenesis is purely coincidental or specific for microinjected rat oocytes.

The finding that large genomic mouse sequences which have been shown to adequately control Cre expression in mouse Cre driver lines likely contain sufficient regulatory information for rat transgenesis suggest that this strategy might be applicable in a general way to generate tissue-specific rat Cre driver lines.

### Functional analysis of Cre-mediated recombination in transgenic TPH2-CreERT2 rats

Novel Cre driver lines need to be functionally assessed for efficiency and tissue-specificity of Cre-mediated recombination. We previously generated a rat Cre reporter line, CAG-loxP.EGFP, which shows CAG-promoter controlled baseline, non-recombined βgal expression and EGFP reporter expression once Cre-mediated recombination of a loxP flanked lacZ STOP cassette has occurred (Schönig et al, in preparation). A major advantage of this strategy is that basal CAG-promoter activity in the tissue of interest can be readily assessed on a cellular level by monitoring βgal expression. Hence, it can be rapidly determined in advance whether CAG-loxP.EGFP rats allow for functional characterization of a new tissue-specific rat Cre driver line. We have characterized the CAG-loxP.EGFP rat Cre reporter line for its utility to monitor Cre-mediated recombination in TPH2-CreERT2 rats. We find strong βgal expression throughout the brain in all examined neuronal populations and in a portion of astrocytes. In particular, CAG-loxP.EGFP rats show abundant monoaminergic βgal expression in the absence of EGFP expression which makes CAG-loxP.EGFP rats suitable to functionally characterize tamoxifen-induced, Cre mediated recombination and background recombination in 5-HT neurons. Using TPH2-CreERT2/CAG-loxP.EGFP double transgenic rats, we functionally validate Cre-mediated recombination, i.e. EGFP expression, in 5-HT neurons of all raphe nuclei upon tamoxifen induction whereas background recombination in vehicle-treated rats was absent. Furthermore, the absence of extraserotonergic EGFP expression in TPH2-CreERT2/CAG-loxP.EGFP rats confirms tissue specificity of the TPH2-CreERT2 driver line.

Serotonergic recombination in tamoxifen-induced TPH2-CreERT2/CAG-loxP.EGFP rats was less efficient than recombination in the previously described mouse TPH2-CreERT2 line [16] (5-HT neuron specific recombination rate: mouse 90% versus rat 77%) while background recombination without tamoxifen was equally low in the rat TPH2-CreERT2 line. The lower recombination efficacy could be due to the integration site of the transgene, its copy number or missing regulatory elements in the mouse *Tph2* sequence which drives CreERT2 expression in the rat brain. Alternatively, the tamoxifen dose or the induction protocol with three single daily injections and only two twice daily tamoxifen injections could potentially result in insufficient nuclear translocation of CreERT2 and thus reduced Cre-mediated recombination. The individual tamoxifen dosage of 40 mg/kg in rats is analogous to 1 mg/injection often used in mice [25]–[27]. In CreERT2 mice, it has been previously shown that the most efficient tamoxifen protocol consists of twice daily tamoxifen injections for 5 consecutive days [25], [27]. The frequency of Cre-mediated recombination in mice decreased considerably with protocols using single daily tamoxifen injections even when the protocol was extended to 10 days [25], [27]. Our initial attempts to apply the mouse protocol of twice daily tamoxifen injections to our transgenic rats failed as the rats did not well tolerate this protocol. Nonetheless, we believe that insufficient tamoxifen-mediated nuclear translocation of CreERT2 is only partially responsible for the found incomplete recombination efficacy since Cre was not expressed at all in some 5-HT neurons.

### Importance of tissue specific rat Cre driver lines for rat transgenesis

Recently, a plethora of new techniques for the modification of the rat genome has been introduced including the development of germline competent embryonic rat stem cells and nuclease based methods [12], [28]–[31]. For the first time, this permits targeted integration of recombinant DNA sequences into the rat genome. In the near future, it is expected that these techniques will be applied to generate conditional loxP-flanked alleles in the rat allowing for spatial and temporal control of gene deletion with tissue-specific rat CreERT2 driver lines. This strategy is of particular importance in order to overcome lethality or induction of compensatory, homeostatic mechanisms or pleiotropy during development, inherent with traditional methods applied in rats such as ENU- or transposon mediated mutagenesis [32]–[34]. Furthermore, the CAG-loxP.EGFP line illustrates how complementary systems for tissue-specific overexpression or knock-down of target genes could be easily implemented. For inducible overexpression, the EGFP reporter cassette would be simply replaced by a candidate gene's cDNA which transcription would only be activated after Cre-mediated recombination. Alternatively, polymerase II controlled microRNAs or sponge/decoy miRNA sequences [35], [36] could be placed downstream of the loxP-flanked lacZ cassette which would allow a Cre-mediated gene knock-down. Apart from tissue specific and inducible overexpression of cDNAs, this technology enables the conditional rescue of gene knockouts, overexpression of mutated gene variants, micro RNA mediated translational repression or the study of microRNA mediated post-transcriptional gene regulation by antagonizing microRNA activity. The TPH2-CreERT2 rat line is also optimally suited for optogenetic manipulations of the serotonergic system with Cre-activated opsin genes delivered to the brain by viruses [37], [38].

As we have demonstrated above, the combination of such Cre activatable “response units” with the TPH2-CreERT2 line will guide modifications specifically to serotonergic neurons.

### Conditional gene manipulations in serotonergic neurons of transgenic rats

Only during the last decade, conditional transgenic mouse tools have been developed to manipulate candidate genes exclusively in 5-HT neurons using the Cre/loxP recombinatorial system [5], [16], [39]. These studies have led to important insights into the physiological role of the 5-HT system [5], [6], [8], [40]–[42]. In contrast, only few publications have addressed the involvement of the 5-HT system in impulsive behavior, cognitive flexibility, decision making, sensitivity to reward, and responsiveness to punishment and aversive signals [9], [43], all functions that have been prominently associated with 5-HT [1], [44], [45]. This comes as no surprise, since the mouse as a model organism for complex behavioral analysis of higher cognitive functions has not been the first choice for most researchers. Because of its size, ease of manipulation and breeding characteristics, the laboratory rat has been the preferred animal model for physiology, pharmacology, toxicology, nutrition, behavior, immunology and neoplasia for many decades while the mouse has emerged as the principal mammal for experimental genetics [46]. Transferring conditional genetic manipulation to the rat would greatly enhance our capabilities to dissect 5-HT functions and its implications for emotions, learning and complex behaviour. With our approach, the advantages of conditional, 5-HT neuron specific genetic manipulation – previously a mouse geneticist's province - can now be studied in the rat with all its amenities.
